# Surgical nuances and placement of subgaleal drains for supratentorial procedures—a prospective analysis of efficacy and outcome in 150 craniotomies

**DOI:** 10.1007/s00701-019-04196-6

**Published:** 2020-01-15

**Authors:** Hussam Aldin Hamou, Konstantin Kotliar, Sonny Kian Tan, Christel Weiß, Blume Christian, Hans Clusmann, Gerrit Alexander Schubert, Walid Albanna

**Affiliations:** 1 Department of Neurosurgery, RWTH Aachen University, Pauwelsstr. 30, 52074 Aachen, Germany; 2 Department of Medical Engineering and Technomathematics, FH Aachen University of Applied Sciences, Aachen, Germany; 3 Department for Medical Statistics and Biomathematics, Medical Faculty Mannheim, Heidelberg University, Mannheim, Germany

**Keywords:** Craniotomy, CSF fistula/leak, Dural closure, Infection, Subgaleal drainage

## Abstract

**Background:**

For supratentorial craniotomy, surgical access, and closure technique, including placement of subgaleal drains, may vary considerably. The influence of surgical nuances on postoperative complications such as cerebrospinal fluid leakage or impaired wound healing overall remains largely unclear. With this study, we are reporting our experiences and the impact of our clinical routines on outcome in a prospectively collected data set.

**Method:**

We prospectively observed 150 consecutive patients undergoing supratentorial craniotomy and recorded technical variables (type/length of incision, size of craniotomy, technique of dural and skin closure, type of dressing, and placement of subgaleal drains). Outcome variables (subgaleal hematoma/CSF collection, periorbital edema, impairment of wound healing, infection, and need for operative revision) were recorded at time of discharge and at late follow-up.

**Results:**

Early subgaleal fluid collection was observed in 36.7% (2.8% at the late follow-up), and impaired wound healing was recorded in 3.3% of all cases, with an overall need for operative revision of 6.7%. Neither usage of dural sealants, lack of watertight dural closure, and presence of subgaleal drains, nor type of skin closure or dressing influenced outcome. Curved incisions, larger craniotomy, and tumor size, however, were associated with an increase in early CSF or hematoma collection (*p* < 0.0001, *p* = 0.001, *p* < 0.01 resp.), and larger craniotomy size was associated with longer persistence of subgaleal fluid collections (*p* < 0.05).

**Conclusions:**

Based on our setting, individual surgical nuances such as the type of dural closure and the use of subgaleal drains resulted in a comparable complication rate and outcome. Subgaleal fluid collections were frequently observed after supratentorial procedures, irrespective of the closing technique employed, and resolve spontaneously in the majority of cases without significant sequelae. Our results are limited due to the observational nature in our single-center study and need to be validated by supportive prospective randomized design.

**Electronic supplementary material:**

The online version of this article (10.1007/s00701-019-04196-6) contains supplementary material, which is available to authorized users.

## Introduction

For most intracranial neurosurgical procedures, there are numerous surgical nuances described in the literature, including variation of surgical access techniques, detailed descriptions and recommendations for dural closure, placement of the bone flap and/or surgical drains, and closure of the skin [2, 7, 36, 43, 47, 53]. The individual choice of technique often depends on surgical experience, personal preference, and departmental standards. Adverse events, however, may still occur: postoperative infections [1, 5, 13, 16, 33, 37], wound dehiscence [4], and leakage of cerebrospinal fluid [31]. These can increase morbidity, prolong hospitalization, and may require surgical and medical intervention, thus increasing socioeconomic costs [19, 34].

Primary watertight dural closure has been suggested for reducing CSF leakage rate, and infections as well as impaired wound healing have been described in the absence of watertight closure [12, 29, 51], but these findings have been questioned by others [7]. Subgaleal fluid collections in general may protract wound healing, but prophylactic placement of drains to address those may also facilitate surgical site infections, secondary wound breakdown, or more serious complications such as infectious destruction of anatomical structures [41]. Active suction may even result in pseudohypoxic brain swelling and death [49].

Currently, there is no clear evidence to support specific recommendations for placement of subgaleal drains, as extremely variable execution of surgery complicates the conduction of randomized studies. While a certain technique is readily justified and implemented, an overly apodictical use of one technique over another may not necessarily result in a superior outcome.

It is the purpose of this single-center observational study to report our experiences of subgaleal drain placement in the context of supratentorial surgeries, utilized upon discretion of the individual treating physician.

## Methods

### Patient selection

For this prospective observational analysis, we consecutively recruited patients undergoing open supratentorial craniotomy between January 2014 and January 2016. Patients with intracranial tumor, intracranial hemorrhage, cerebrovascular pathology, or closed traumatic brain injury referred to our neurosurgical department were included. Patients with previous craniotomy or those requiring decompressive craniectomy, cases with previous or ongoing radiation or chemotherapy, and those in need of CSF diversion or drainage of an intracranial abscess were excluded. As part of this observational study and in addition to demographic data, individual surgical nuances (see below) were permitted and recorded.

Ethics approval was given by the local ethics committee (ID 082013), and written consents for study inclusion were obtained from all patients or their authorized representatives.

### Demographic data

We collected demographic data, sex, age, weight, size, and body mass index (BMI). The following comorbidities were recorded to identify any relevant confounding factors for CSF leakage, infection, or impaired wound healing such as diabetes mellitus, arterial hypertension, anemia/thrombocytopenia, coronary heart disease, dermatitis, preoperative anticoagulation, and smoking.

### Operative methods

All craniotomies were performed in the operating room using a laminar air flow system. Patients received compression stockings for prophylaxis of deep venous thrombosis, and subcutaneous heparin was started 24 h after surgery. The following prophylactic antibiotics were administered to all patients: cefuroxim (1.5 g), or—in case of penicillin allergy—clindamycin (600 mg) approximately 30 min before the start of the operation, and repeated every 3 h intraoperatively. Postoperative prophylactic antibiotics were not given. To prepare the scalp for incision, the scalp was shaved (1–2 cm width along the marked incision), prepared by alcohol and iodine derivatives (Braunoderm®, skin antiseptic, B. Braun Melsungen AG, Germany), and the surgical site was covered with a sterile transparent foil. At the end of the operation, meticulous hemostasis was ensured, followed by extradural irrigation (iodine and saline solution). Based on the individual surgeon’s preference, a surgical silicone drain was placed subgalealy in the subgaleal drains group, then tunneled and delivered through a separate stab incision and attached to the closed drain system to gravity (Robdrain®, 12 CH., outer diameter 4 mm, length 100 cm, B. Braun Melsungen AG, Melsungen, Germany). Standard wound closure was then performed by interrupted single sutures (subgaleal layer, subcutaneous layer), followed by closure of the skin (sutures or staples). Compressive dressings were used at the surgeon’s discretion.

Following the surgery, the respective surgeon recorded all relevant technical aspects: type (curved/straight) and length of incision, technique of dural and skin closure (stitched or stapled), placement of subgaleal drains, and postoperative compressive dressing. Craniotomy reconstruction using titanium clamps (Craniofix®) or polyester sutures (Mersilene®) and intraoperative antiseptic irrigation (hydrogen peroxide or 1% povidone-iodine) were also documented. Intraoperative iatrogenic complications such as accidental opening of frontal sinuses or mastoid cells were also recorded. Craniotomy size was calculated with the maximum anterio-posterior (AP) and maximum cranio-caudal (CC) diameter with the consideration of the elliptic shape of craniotomies according to the following formula: π × (CC/2) × (AP/2).

For comparative analysis, patients were then primarily stratified according to the presence or absence of non-suction subgaleal drains (+drain, −drain) placed during closure. For comparative analysis, patients were then stratified according to the median of the craniotomy size (≥ or < 27 cm^2^) and length of the scalp incision (≥ or < 15 cm).

### Postoperative management and outcome

The surgical site was examined regularly with initial daily dressing changes. Compression bandage—if present—was removed 24 h postoperatively. Gravity drains were removed under sterile conditions when the flow rate was less than 100 ml in the previous 24 h, with all drains being removed after a maximum of 48 h, closing the exit site with a single suture.

The following outcome parameters were recorded: presence of early periorbital edema graded as moderate or severe (eye opening impeded or not possible), presence of subgaleal swelling graded as moderate, or severe (including bulging and firm collections) at the time of discharge (early follow-up) and after 6 weeks (late follow-up), any impairment of wound healing (up to 6 weeks postoperatively) or any need for operative revision. Provided regular, primary wound healing, sutures, or staples were routinely removed on the 7th–9th postoperative day. In cases of wound breakdown (dehiscence of cutaneous layer, visibility of subcutaneous tissue or bone, hematoma, or cerebrospinal fluid leakage through the skin), the indication for operative revision was evaluated on an individual basis. Infection was defined as any of the following: evidence of a purulent wound, meningitis (verified by lumbar puncture), intracerebral abscess, or wound healing disorder in conjunction with increased inflammatory parameters (early or late follow-up).

Neurological examinations were performed daily and at time of discharge. The subjective pain level was assessed using the visual analogue scale (VAS).

### Statistical analysis

Risk factor analysis was performed in two steps. Starting univariate logistic regression model, we identified variables that may influence outcome parameters. This step was used as a model building process, based on the decision rule, that factors showing a *p* value of less or equal to 0.1 are used in the corresponding multivariate logistic regression model. In this last step, factors were assessed as significant, if the corresponding *p* value fell below the 5% margin. We reported our results by *p* values, odds ratios and corresponding 95% confidence intervals (Cl). Quantitative variables are summarized by mean and standard deviations (SD) and categorical data by absolute frequencies (*n*) and percentages (%). In selected cases, chi square test was performed. All analyses were performed using IBM® SPSS® Statistics V22.0 (IBM, Chicago, Illinois, USA). Statistical significance was set at *p* < 0.05, and statistical results with *p* < 0.1 were accepted as a trend.

## Results

### Demographic and surgical data

A total of 150 craniotomies were performed for the following pathologies: tumor (74.7%; *n* = 112), hematoma (subdural 6.7%, *n* = 10 or intraparenchymal 4.0%, *n* = 6), cerebrovascular disease (aneurysm, cavernoma, or arteriovenous malformations, 13.3%; *n* = 20), and unspecific lesions (1.3%, *n* = 2). 39.3% of patients were male (*n* = 59), mean age of all cases was 59.1 ± 14.8 years.

The incision was performed in a straight line in 49.3% of patients and curved in the remaining 50.7%, with an average length of 16.7 ± 5.7 cm (median 15 cm), resulting in short incisions in 57.7% (13.0 ± 2.9 cm) and longer incisions in 42.2% (21.7 ± 4.8 cm). We recorded an average size of craniotomy of 31.4 ± 20.7 cm^2^ (median 27 cm^2^), resulting in 52.3% of smaller craniotomies (18.0 ± 6.6 cm^2^) and 47.7% of larger craniotomies (46.1 ± 20.9 cm^2^).

Intraoperative ventricle opening occurred in 14% (*n* = 21); watertight closure with or without dural sealants was achieved in 77.3% (*n* = 116), non-watertight dural closure in 22.7% (*n* = 34).

The majority of skin closures was performed using staples (70%), and tendentially more often in cases with longer skin incisions (*p* = 0.069), but not with larger craniotomies (*p* = 0.383). Compressive dressing was used in 33.3% of cases and did not depend on the length of incision or the size of the craniotomy (*p* = 0.889, *p* = 0.274, data not shown).

For the subgroup analysis, we identified 112 patients undergoing surgery for neoplastic disease; this group was further stratified according to neoplastic entity, size, and localization (Suppl. Table 1).

### Association of subgaleal drainage and other surgical variables

Subgaleal drains were used in 42% of cases. Patients with and without drains (+drain vs −drain) were comparable in terms of basic demographic data and clinical features. Patients with longer incisions and larger craniotomies received drains significantly more often (incision > 15 cm: +drain 67.7% vs −drain 24.1%, *p* < 0.0001; craniotomy > 27 cm^2^: +drain 62.9% vs −drain 36.8%, *p* < 0.01). With watertight dural closure with or without sealants, drains were placed significantly more often (+drain 88.9% vs −drain 69.0%, *p* < 0.01); wounds were significantly more often closed with stables, when a drain was used (+drain 87.3% vs −drain 57.5%, *p* < 0.0001). Patients with subgaleal drains received compressive dressings more frequently (+drain 47.4% vs −drain 23.5%, *p* < 0.01) (Table 1).

**Table 1:** Surgical variables for all patients, as well as in the presence and absence of subgaleal drains (+drain, −drain)

Surgical variables		Total	+Drain	−Drain	*p* value
		*n* (%) or mean ± SD			
Access	Curved: straight incision	76 (50.7%), 74 (49.3%)	43 (68.3%), 20 (31.7%)	33 (37.9%), 54 (62.1%)	*p* < 0.0001
	Longer incision ≥ 15 cm Shorter incision < 15 cm	63 (42.2%), 86 (57.7%)	42 (67.7%), 20 (32.3%)	21 (24.1%), 66 (75.9%)	*p* < 0.0001
	Larger craniotomy ≥ 27 cm^2^: Smaller craniotomy < 27 cm^2^	71 (47.7%), 78 (52.3%)	39 (62.9%), 23 (37.1%)	32 (36.8%), 55 (63.2%)	*p* < 0.01
Intraoperative ventricle opening		21 (14%)	8 (12.7%)	13 (14.9%)	ns
Closure	non-watertight dural closure: watertight dural closure with or without sealants	34 (22.7%), 116 (77.3)	7 (11.1%), 56 (88.9%)	27 (31.0%), 60 (69.0%)	*p* < 0.01
	suture: staples	45 (30%), 105 (70%)	8 (12.7%), 55 (87.3%)	37 (42.5%), 50 (57.5%)	*p* < 0.0001
	compressive dressing	46 (30.7%)	27 (42.9%)	19 (21.8%)	*p* < 0.01

*P*-values are calculated for surgical variables after stratifying in two groups (+drain vs −drain). Patients with curved incisions, longer incisions, and larger craniotomies received drains significantly more often. With watertight dural closure with or without sealants, drains were placed significantly more often; wounds were significantly more often closed with stables, when a drain was used. Patients with subgaleal drains received compressive dressings more frequently

+drain, patient with subgaleal drainage; −drain, patient without subgaleal drainage; *ns*, not significant; *SD*, standard deviation

### Postoperative course and outcome parameter

Early periorbital edema was observed in 10% (*n* = 15) of cases. 34.8% (*n* = 55) of patients developed early subgaleal swelling (moderate and severe), which persisted in 2.3% (*n* = 3) at late follow-up. Two of these cases had to undergo operative revision. In 3.3% (*n* = 5) of cases, wound healing was impaired, and a total of 6.7% (*n* = 10) had to undergo any kind of operative procedure (puncture, secondary stitches, or operative revision). Adequate pain control was achieved in 90.4% of patients in the early follow-up and in 97.5% in the late follow-up.

### Association of subgaleal drainage and outcome

The effect of drainage on recorded outcome parameters was analyzed using the binary logistic regression model. The incidence of periorbital edema (moderate or severe), subgaleal swelling (moderate and severe, early and late), impaired wound healing (until late follow-up), non-adequate pain control (early and late f/u), and need for operative revision was not affected by the presence of subgaleal drains (*p* = 0.115, *p* = 0.515, *p* = 0.395, *p* = 0.927, *p* = 0.927, *p* = 0.586, *p* = 0.388, *p* = 0.895) (Table 2). Early pain control (VAS 0–4) was acceptable in both groups (1st day postoperative: +drain 91.2% vs −drain 91.8%, *p* = 0.607; 3rd day postoperative: +drain 88.6% vs −drain 89.8% *p* = 0.564) (data not shown in tables). Tumor entity did not influence the decision to the placement of subgaleal drains. After additional subgroup analysis, larger tumor size was significantly associated with higher infection rate (*p* = 0.043) with more infections related to metastasis (*p* = 0.017) (Suppl. Table 2). In this context, subgaleal drains did not influence the infection rate.

**Table 2:** Univariate regression analysis of outcome parameters with and without drainage

Outcome parameter	Total	+Drain	−Drain	Logistic regression		
	*n* (%) or mean ± SD			OR	95% CI	*p* value
Periorbital edema (early; moderate or severe)	9 (10.0%)	6 (16.2%)	3 (5.7%)	0.310	0.072–1.330	ns
Subgaleal swelling (early)	55 (36.7%)	25 (39.7%)	30 (34.5%)	0.800	0.409–1.565	ns
Subgaleal swelling (late)	3 (2.3%)	2 (3.6%)	1 (1.3%)	0.349	0.031–3.945	ns
Impaired wound healing	5 (3.3%)	2 (3.2%)	3 (3.4%)	1.089	0.177–6.718	ns
Pain, VAS 5–10 (early)	8 (9.6%)	4 (11.8%)	4 (8.2%)	0.667	0.155–2.873	ns
Pain, VAS 5–10 (late)	3 (2.5%)	2 (4.0%)	1 (1.4%)	0.343	0.030–3.888	ns
Need for operative revision	10 (6.7%)	4 (6.3%)	6 (6.9%)	1.093	0.295–4.045	ns
Infection	11 (7.3%)	4 (6.3%)	7 (8.0%)	1.291	0.361–4.613	ns

The effect of drainage on recorded outcome parameters was analyzed using the binary logistic regression model. Outcome parameter at the time of discharge was decelerated as early follow-up; after 6 weeks, as late follow-up. The incidence of periorbital edema (moderate or severe), subgaleal swelling (moderate and severe, early and late), impaired wound healing (until late follow-up), non-adequate pain control (early and late f/u), need for operative revision, and infection was not affected by the presence of subgaleal drains. Infection was defined as any of the following: evidence of a purulent wound, meningitis (verified by lumbar puncture), intracerebral abscess, or wound healing disorder in conjunction with increased inflammatory parameters (early or late follow-up).

*CI*, confidence interval**;** +drain, patient with subgaleal drainage; −drain, patient without subgaleal drainage; *ns*, not significant; *OR*, odds ratio; *SD*, standard deviation; *VAS*, visual analog scale

### Effect of other surgical variables on outcome

To identify the effect of other surgical variables on outcome, the same regression model approach was selected as described above. Early periorbital edema was not affected by any of the surgical parameter (length of incision, shape of the incision, craniotomy size, intraoperative ventricle opening, type of dural or wound closure, and presence of compressive dressing). Subgaleal swelling (early f/u) occurred more frequently in cases with longer wound incision (OR 1.104, 95% CI 1.035–1.178, *p* = 0.003), curved wound incision (OR 3.821, 95% CI 1.873–7.797, *p* < 0.0001) and larger craniotomy size (OR 1.040, 95% CI 1.018–1.062, *p* < 0.001). Larger tumor size was associated with an increase in early CSF or hematoma collection (*p* < 0.01) but lost his significant in the late follow-up (*p* = 0.365).

Based on the corresponding multivariate logistic regression model, the significantly higher rate of subgaleal swelling at the early f/u was only linked to larger craniotomy size (OR 1.032, 95% CI 1.006–1.059, *p* < 0.05) and selection of curved incisions (OR 0.336, 95% CI 0.156–0.723, *p* < 0.01), but was not related to the incision length (OR 1.008, 95% CI 0.926–1.097, *p* = 0.859), (data not shown in tables).

Late clinical follow-up was available in 88% of patients. At late follow-up, subgaleal swelling persisted in patients with larger craniotomies (OR 1.040, 95% CI 1.001–1.080, *p* = 0.047). At the early and late follow-up, postoperative infections were found in 7.3% of cases and were not affected by any of the recorded surgical variables. No further relevant differences were observed, and this was confirmed also when only including patients undergoing surgery for neoplastic diseases (Suppl. Table 2).

## Discussion

According to the literature, postoperative complications after craniotomies such as intra- or extracranial bleeding, leakage of cerebrospinal fluid (CSF) [32, 40], impaired wound healing, and frank wound infection are infrequently observed, but nevertheless increase morbidity rate [1, 20, 29, 37] as well as socioeconomic costs after surgeries [1, 37] considerably. Subgaleal hematomas and fluid collections as such occur more often after cranial surgery and are believed to increase both patient discomfort as well as the overall postoperative complication rate. Historically, the use of subgaleal drains was first described by Ames et al. [3]. Drains may decrease the rate of postoperative fluid collections and other complications, and—as a consequence—are employed by several surgeons, while the objective benefit is still unclear [8, 30].

### Effect of prophylactic antibiotics on clinical course

As recommended by the clinical practice guidelines in surgery, we routinely administer prophylactic preoperative antibiotics in all craniotomies [10]. While routine administration of postoperative antibiotics is common practice in many institutions, the risk of surgical site infection (SSI) remains unaffected [6, 22, 24, 27, 46].

The use of postoperative antibiotics did not belong to the standard operating procedure in our clinic as they seem to be associated with drawbacks including allergic reaction, gastrointestinal upset, infection with *Clostridium difficile*, or other resistant organisms and can increase in-hospital costs [18, 23].

### Possible complications and side effects after placement of subgaleal drains

The placement of subgaleal drains with or without suction is often dependent on the experiences or preferences of the surgeons and/or intraoperative findings [11], the underlying hypothesis being the reduction subgaleal collection, thus avoiding wound tension, pain, and possibly (super) infection. However, previous studies suggest that the complication rate is not reduced after supratentorial craniotomies, but can include bradycardia [15, 28, 48], asystole [9, 52], bleeding from the superior sagittal sinus [35], epidural hematomas [11, 41, 49], pseudohypoxic brain swelling with fatal courses [49], and sudden intracranial pressure drop with rupture of a partially clipped aneurysm [39]. While in most of the abovementioned, catastrophic incidences, subgaleal drainages on suction were used, open gravity drainage can result in comparable suction effect when placed below body level. Other negative aspects of using subgaleal drains include increased economic costs, the necessity of and discomfort when removing drains, headache, and anxiety. Fresh blood clots may even impede the functionality of drains, with persistent subgaleal fluid collections. In the context of spine surgery or orthopedic operations, placement of drains does not seem to prevent postoperative epidural bleeding and overall infection rate, though an increase in patient discomfort is noted [14, 38, 45, 54]. After abdominal surgery (laparotomy), Higson et al. found a duplication of infection using non-aspirating drainage, whereas other studies did not confirm this association [25]. Drains can generally lead to a reduction in pain after surgeries such as total knee arthroplasty, but the results in this randomized prospective study were only confirmed in the early-term but not long-term follow-up [17]. However, in considering the postoperative pain in other disciplines, Schietroma et al. found that routine drain placement after thyroidectomy is not necessary, and avoiding drainage was associated with less postoperative pain [44]. The controversial results in between the trials mentioned above lead ultimately to different conclusions which are possibly subject-specific.

Due to the conflicting results and findings of previous studies and the strong personal belief of treating physicians, it was the purpose of this observational study to determine the effect—if any—of surgical nuances and preferences (type of wound closure and use of drains) on the postoperative course after supratentorial craniotomy.

### Effect of subgaleal drains on recorded outcome parameters

In our cohort, the infection rate was 7.3%, corresponded well with findings of other groups (infection rate between 1 and 11%) [1, 16, 33, 37] and was comparable in patients with and without drains; further surgical parameters did not affect the infection rate. The presence of drains was not associated with a difference in wound healing or the need for operative revision but also did not decrease periorbital edema or subgaleal swelling nor the postoperative perception of pain, as initially hypothesized.

### Effect of other surgical variables on outcome parameters

Regarding supratentorial craniotomies, our results are in line with other studies that the watertight dural closure is not necessarily associated with an increased rate of complications [7, 42]. Even in the case of larger surgeries such as decompressive craniectomies, complications related to non-watertight dural closure seem to be negligible [21, 50], and may be more related to quality and technique of skin closure. We did not observe differences in outcome caused by skin closure techniques using staples or stitches. However, the workgroup Abu Hamed et al. described significantly increased infection rates related to the use of staples instead of sutures [1], though possibly inherent to study design and not reproduced in our observational analysis. John et al. 2017 [26] reported a negative influence on outcome in patient with ventricle opening during tumor surgery, an effect that was not observed in our cohort. A possible explanation for this discrepancy may be the heterogeneity of our cohort and the limited number of patients with intracranial tumor surgeries with intraoperative ventricle opening.

It can be speculated that placement of a drain and the use of compressive dressings are more common in younger, possibly less experienced surgeons under the assumption of avoiding postoperative complications. Though our analysis did not include stratification by experience and years of practice, the use of drains according to personal preference appears acceptable, as the associated complication rate was not higher, even in the presence of larger and possibly more hazardous craniotomies.

### Limitations

Our study is limited due to the observational nature of the design. The monocentric, non-randomized analysis does not readily allow generalization or extrapolation to other centers. There is an inherent selection bias, and we can only confirm that accommodating the individual surgeon’s preference/nuance in our center resulted in comparable outcome.

In general, subgaleal drains can be placed based on the preference of surgeons whereby the advantage of drains could not be ensured by the present study for any of the recorded subjective and objective outcome parameters. At the same time, recorded operative nuances do not appear to play a major role in determining outcome, thus it is questionable, whether larger prospective studies are justified.

## Conclusions

Permissive attitudes towards surgical nuances and preferences—including the use of subgaleal drains and differences in dural closure—can result in comparable outcome after supratentorial craniotomies.

## Electronic supplementary material

ESM 1 (DOCX 36 kb)
