# Supplementary material for: Surgical nuances and placement of subgaleal drains for supratentorial procedures—a prospective analysis of efficacy and outcome in 150 craniotomies
Source: Acta Neurochir (Wien). 2020 Jan 15;162(4):729–36. doi: 10.1007/s00701-019-04196-6 (PMC7066100; doi:10.1007/s00701-019-04196-6)
Supplement: Supplementary file 1 — (DOCX 36 kb) [file 701_2019_4196_MOESM1_ESM.docx]

**Supplemental Table 1**

Tumor entity and the relation to tumor size. LGG, low grade glioma; HGG, high grade glioma

| **parameter** | **total** | **tumor size**  **median**  **[1. quartile-3. quartile]** |
| --- | --- | --- |
| tumor | 112 (100%) | 50.0 [18.2-89.7] cm^3^ |
| meningioma | 35 (31.3%) | 37.4 [15.0-73.3] cm^3^ |
| convexity meningioma | 20 (17.9%) | 36.1 [17.1-68.4] cm^3^ |
| convexity meningioma ≥36 cm^3^ | 10 (8.9%) | 63.6 [48.7-249.2] cm^3^ |
| convexity meningioma <36 cm^3^ | 10 (8.9%) | 19.2 [3.5-34.8] cm^3^ |
| metastasis | 24 (21.4%) | 53.4 [21.4-79.4] cm^3^ |
| subcortical  metastasis | 12 (10.7%) | 9.0 [5.8-76.4] cm^3^ |
| subcortical  metastasis ≥9 cm^3^ | 6 (5.4%) | 73.5 [11.7-107.0] cm^3^ |
| subcortical  metastasis <9 cm^3^ | 6 (5.4%) | 6.1 [3.1-8.5] cm^3^ |
| glioma | 46 (41.1%) | 62.0 [37.2-123.4] cm^3^ |
| LGG | 9 (8%) | 44.0 [28.4-103.9] cm^3^ |
| HGG | 37 (33%) | 63.6 [37.8-123.5] cm^3^ |
| others (colloid cyst, hemangioma, hemangioblastoma) | 7 (6.3%) | 2.9 [1.0-20.2] cm^3^ |

| **outcome parameter** | **variable** | **logistic regression** | | |
| --- | --- | --- | --- | --- |
|  |  | OR | 95% CI | p-value |
| **periorbital edema**  **(early; moderate or severe)** | meningioma | 0.952 | 0.081-11.134 | 0.969 |
| **n=3** | metastasis | - | - | 0.999 |
|  | glioma | 0.361 | 0.031-4.197 | 0.416 |
|  | others (colloid cyst, hemangioma, hemangioblastoma) | - | - | 0.999 |
|  | tumor size | 1.001 | 0.984-1.018 | 0.943 |
| **subgaleal swelling**  **(early)** | meningioma | 1.596 | 0.672-3.789 | 0.289 |
| **n=40** | metastasis | 2.509 | 0.858-7.343 | 0.093 |
|  | glioma | 0.293 | 0.131-0.658 | **0.003** |
|  | others (colloid cyst, hemangioma, hemangioblastoma) | 3.545 | 0.411-30.6 | 0.249 |
|  | tumor size | 1.009 | 1.003-1.015 | **0.006** |
| **subgaleal swelling**  **(late)** | meningioma | 0.239 | 0.021-2.732 | 0.249 |
| **n=3** | metastasis | 0.538 | 0.047-6.230 | 0.620 |
|  | glioma | - | - | 0.999 |
|  | others (colloid cyst, hemangioma, hemangioblastoma) | - | - | 0.999 |
|  | tumor size | 1.006 | 0.993-1.020 | 0.365 |
| **impaired wound healing** | meningioma | 1.378 | 0.138-13.739 | 0.784 |
| **n=4** | metastasis | - | - | 0.999 |
|  | glioma | 2.143 | 0.216-21.272 | 0.515 |
|  | others (colloid cyst, hemangioma, hemangioblastoma) | 2.165 | 0.219-19.250 | 0.249 |
|  | tumor size | 1.005 | 0.992-1.017 | 0.451 |
| **need for operative revision** | meningioma | 0.538 | 0.135-2.140 | 0.379 |
| **n=9** | metastasis | - | - | 0.999 |
|  | glioma | 0.861 | 0.218-3.395 | 0.830 |
|  | others (colloid cyst, hemangioma, hemangioblastoma) | 0.495 | 0.053-4.632 | 0.538 |
|  | tumor size | 1.003 | 0.994-1.013 | 0.457 |
| **Pain, VAS 5-10 (early)** | meningioma | 0.789 | 0.122-5.119 | 0.804 |
| **n=5** | metastasis | 0.173 | 0.024-1.252 | 0.082 |
|  | glioma | 3.250 | 0.342-30.884 | 0.305 |
|  | others (colloid cyst, hemangioma, hemangioblastoma) | - | - | 0.999 |
|  | tumor size | 1.008 | 0.996-1.019 | 0.191 |
| **Pain, VAS 5-10 (late)** | meningioma | - | - | 0.999 |
| **n=2** | metastasis | - | - | 0.998 |
|  | glioma | - | - | 0.998 |
|  | others (colloid cyst, hemangioma, hemangioblastoma) | - | - | 0.999 |
|  | tumor size | 0.672 | 0.336-1.345 | 0.262 |
| **Infection** | meningioma | 0.205 | 0.474-32.810 | 0.205 |
| n=9 | metastasis | 0.181 | 0.044-0.738 | **0.017** |
|  | glioma | 1.433 | 0.340-6.051 | 0.624 |
|  | others (colloid cyst, hemangioma, hemangioblastoma) | - | - | 0.999 |
|  | tumor size | 1.009 | 1.000-1.017 | **0.043** |

**Supplemental Table 2**

The effect of tumor entity and tumor size on outcome parameters was analyzed using the binary logistic regression model. Outcome parameter at the time of discharge was decelerated as early follow-up; after six weeks, as late follow-up. Subgaleal swelling (early f/u) occurred more frequently in cases with larger tumor size. Larger tumor size was significantly associated with a higher infection rate with more infections related to metastasis without any association with the placement of subgaleal drains. No further relevant differences were observed. Infections were defined as any of the following: evidence of a purulent wound, meningitis (verified by lumbar puncture), intracerebral abscess, or wound healing disorder in conjunction with increased inflammatory parameters (early or late follow up). CI, confidence interval; +drain, patient with subgaleal drainage; -drain, patient without subgaleal drainage; ns, not significant; OR, Odds Ratio; SD, standard deviation; VAS, visual analog scale.
